# Recent Advances and Perspectives on Field‐Effect Transistors for Artificial Visual Neuromorphic Systems

**DOI:** 10.1002/advs.202518193

**Published:** 2026-02-27

**Authors:** Liu Yaqian, Lang Menghua, Xu Yihang, Zhang Manyu, Lei Chunkang, Lu Xiaozhou, Dou Yi, Wang Lingli, Jiang Liying, Hu Yuanyuan, Chen Huipeng, Jiang Lang

**Affiliations:** ^1^ School of Electronics and Information Zhengzhou University of Light Industry Zhengzhou China; ^2^ Academy For Quantum Science and Technology Zhengzhou University of Light Industry Zhengzhou China; ^3^ Henan Key Laboratory of Information Functional Materials and Sensing Technology Zhengzhou University of Light Industry Zhengzhou China; ^4^ Changsha Semiconductor Technology and Application Innovation Research Institute College of Semiconductors (College of Integrated Circuits) Hunan University Changsha China; ^5^ Institute of Optoelectronic Display National & Local United Engineering Lab of Flat Panel Display Technology Fuzhou University Fuzhou China; ^6^ School of Chemical Engineering Hebei University of Technology Tianjin China; ^7^ Beijing National Laboratory For Molecular Sciences Key Laboratory of Organic Solids Institute of Chemistry Chinese Academy of Sciences Beijing China

**Keywords:** artificial visual neuromorphic systems, field effect transistors, synaptic transistors, visual perception

## Abstract

The exponential growth of data has exposed the inherent bottlenecks of the von Neumann architecture—specifically its limited computational efficiency and high energy consumption—necessitating an urgent shift toward innovative hardware solutions. Biological perception systems, particularly the human visual system, serve as a premier model for highly integrated, energy‐efficient, and multimodal processing, providing a critical blueprint for the future of intelligent computing. Field‐effect transistors (FETs) have emerged as a leading platform for visual neuromorphic systems, leveraging their exceptional optoelectronic tunability, mechanical flexibility, and low‐power operation. This review provides a comprehensive overview of FET‐based visual neuromorphic systems, covering semiconductor material selection, fundamental device architectures, and governing operational principles. Then, the critical role of these devices in emulating biological visual functions is detailed. Finally, the prevailing technical challenges and future development prospects for FET‐mediated perception are discussed. This work aims to provide essential insights into the design of the next generation of artificial visual neuromorphic systems and bio‐inspired electronics.

## Graphical abstract

This review presents a comprehensive overview of FET‐based visual neuromorphic systems, covering their semiconductor materials, core device architectures, and operating mechanisms. It further reviews their implementation in emulating biological visual functions, addresses current technological challenges, and outlines future development directions. The work aims to inform the design of next‐generation bio‐inspired artificial vision hardware.

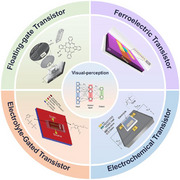

## 1 Introduction

The rapid advancement of artificial intelligence (AI) and machine learning (ML) has driven explosive growth in data volume, imposing significant challenges on the conventional von Neumann architecture. Owing to the physical separation of computing and memory units, this architecture requires frequent data transfers between the central processing unit and memory unit, resulting in high latency, substantial energy consumption, and bandwidth limitations [1, 2, 3, 4, 5, 6, 7, 8, 9]. Noticeably, the human brain integrates storage and computation within densely interconnected networks of neurons and synapses, enabling highly efficient parallel processing of large amounts of information [10, 11, 12, 13]. Meanwhile, the biological visual system, in particular, processes complex visual stimuli seamlessly and with exceptionally low energy consumption (1–100 fj) [14]. Inspired by this neurobiological architecture, artificial neuromorphic computing seeks to emulate the brain's neural networks by co‐locating memory and processing. This design enables the simultaneous execution of tasks such as data transmission, processing, learning, and memory within the same physical unit [15]. By mitigating the need for constant data shuttling, the artificial visual neuromorphic system addresses key limitations of conventional vision sensors, promising substantial improvements in processing speed, energy efficiency, and adaptability to complex, dynamic environments [16, 17, 18, 19].

**FIGURE 1:**
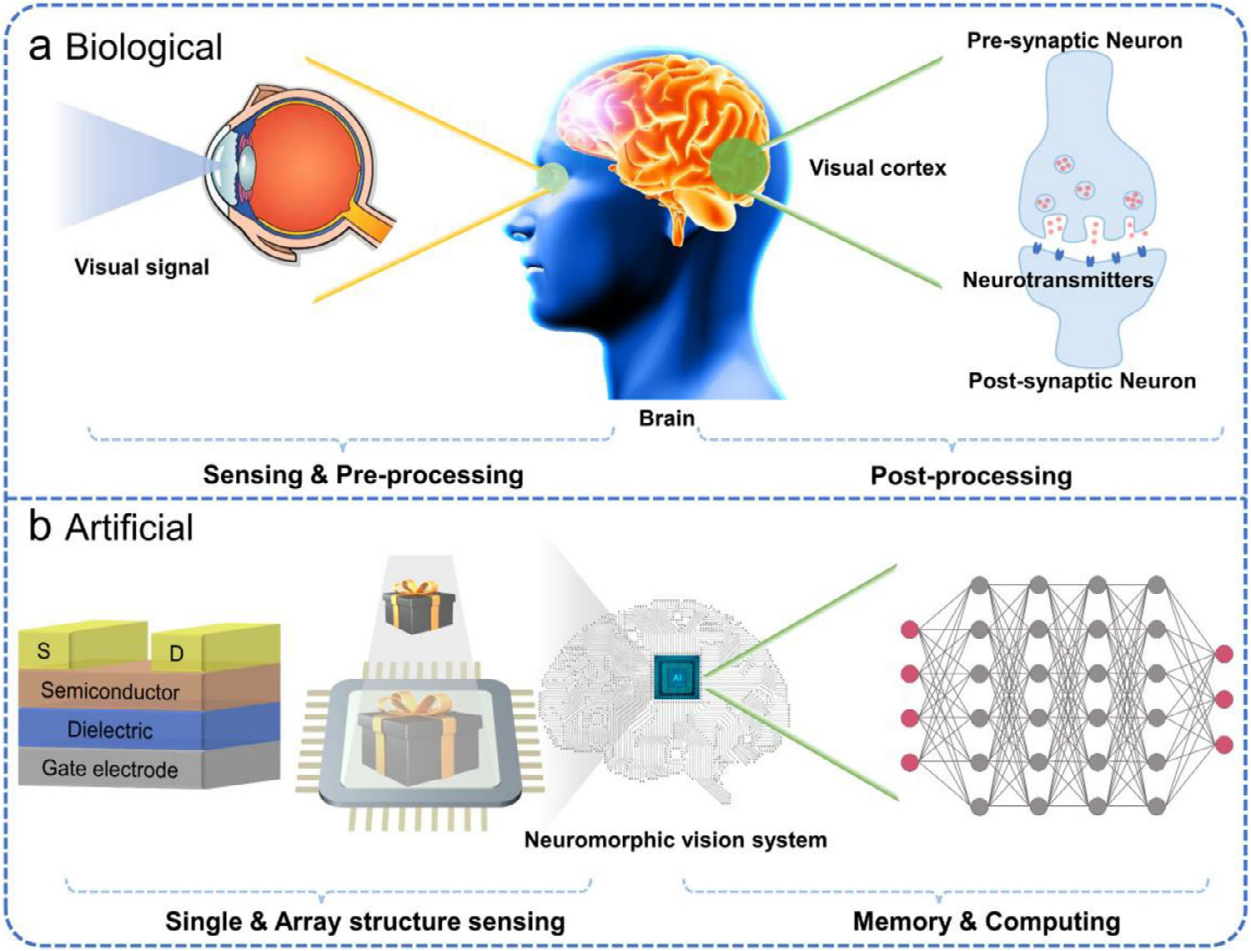
Schematic comparison between biological and artificial perception systems. (a) The biological visual system operates through the conversion of external stimuli by the eyes, followed by signal propagation to the visual cortex for processing along specialized neural pathways mediated by synaptic interactions. (b) In artificial visual perception systems, external optical signals are transduced and retained by single‐transistor devices or integrated arrays. Subsequent computation, such as feature extraction and pattern recognition, is typically performed via deep learning algorithms.

Artificial visual neuromorphic systems operate by detecting and storing external optical signals with photosensitive synaptic devices. Subsequently, computational processing is performed via deep learning algorithms or array‐based architectures (Figure 1). Typical artificial synaptic devices function as biomimetic signal‐processing units, including two‐terminal memristors and three‐terminal transistors. Two‐terminal memristors [20, 21, 22, 23, 24, 25, 26], which lack a gate electrode and possess a compact, simple structure that favors high‐density integration. Among these, phase‐change memory achieves multi‐level storage by switching the phase‐change material between crystalline and amorphous states, offering advantages such as high operating speed, low power consumption, and multi‐state programmability [27]. Resistive random‐access memory encodes information through resistance changes, typically enabled by the formation and dissolution of conductive filaments (e.g., oxygen vacancies), allowing reversible switching between high‐ and low‐resistance states [28]. Magnetic memory, meanwhile, relies on the spin‐transfer torque effect, in which spin‐polarized electrons interact with a magnetic free layer to drive domain wall motion or magnetization reversal. This modulates the resistance state of magnetic tunnel junctions, enabling non‐volatile control of electrical conductivity [29]. However, although such devices can emulate basic synaptic weight modulation, they often struggle to simultaneously support high‐speed signal transmission and complex learning tasks. This limitation stems from insufficient coupling efficiency between signal transmission and learning mechanisms, hindering their ability to fully reproduce the dynamic plasticity of biological synapses.

In comparison, three‐terminal field‐effect transistors (FETs) offer distinct advantages for neuromorphic computing by enabling precise, independent control of channel conductance through the gate terminal [30, 31, 32, 33, 34, 35, 36, 37]. This three‐terminal configuration allows for the decoupled modulation of synaptic weights, facilitating parallel information processing and ultra‐low‐power computing at the edge. By leveraging the gate‐tunable nature of the channel, FETs can effectively emulate complex biological synaptic functions and plasticity, including short‐term plasticity (STP), long‐term plasticity (LTP), paired‐pulse facilitation (PPF), and spike‐timing‐dependent plasticity (STDP) [17, 38]. To achieve these functionalities, various transistor architectures have been developed, most notably floating‐gate FETs (FGFETs), ferroelectric FETs (FeFETs), organic electrochemical FETs (OECTs), and electrolyte‐gated FETs (EGTs). Beyond architectural diversity, the performance of these devices has been further enhanced through the integration of advanced semiconductor materials, such as 2D materials, metal oxides, and hybrid semiconductors. Recently, these FET‐based platforms have evolved to process optical signals directly, mimicking the dual neuronal and synaptic behaviors of the biological visual system. These advancements have positioned FET‐based neuromorphic hardware as a cornerstone for visual perception, demonstrating significant potential in complex pattern recognition and adaptive learning.

**FIGURE 2:**
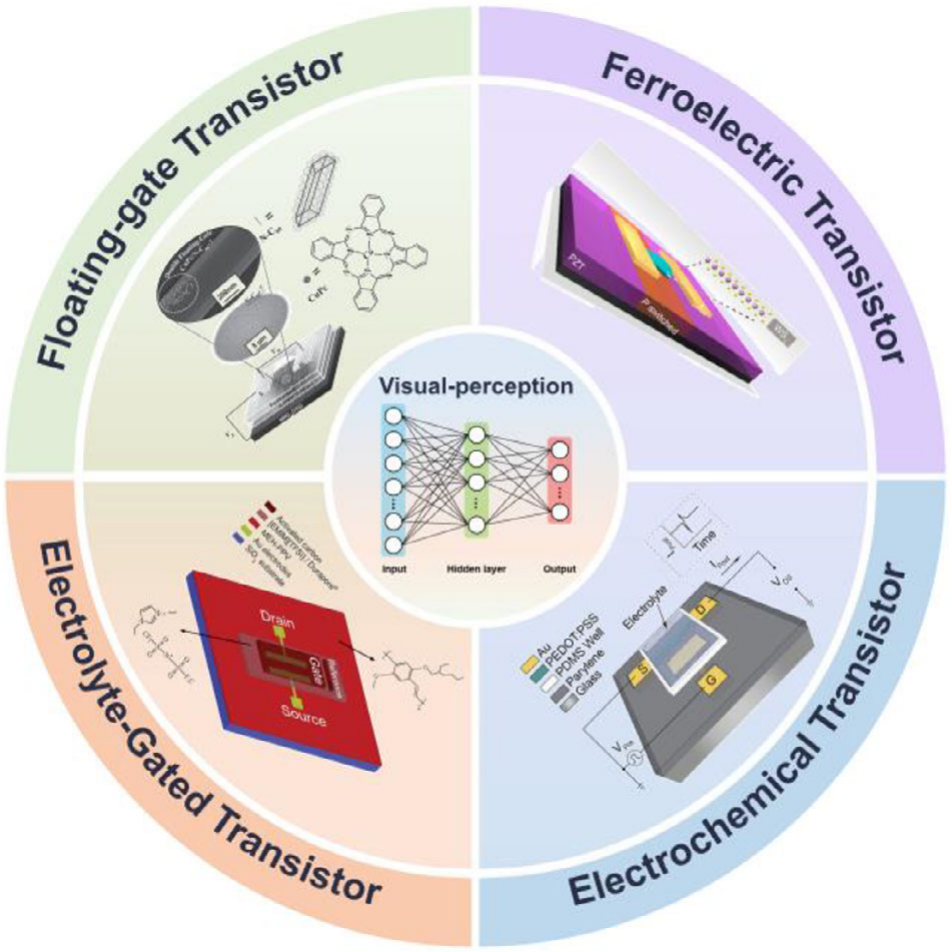
Schematic illustration of FET devices based on different structures. Floating‐Gate Field‐Effect Transistor, Ferroelectric Field‐Effect Transistor, Organic Electrochemical Field‐Effect Transistor, Electrolyte‐Gated Field‐Effect Transistors. Reproduced with permission [89]. Copyright 2014, Wiley. Reproduced with permission [193]. Copyright 2020, American Chemical Society. Reproduced with permission [112]. Copyright 2015, Wiley. Reproduced with permission [120]. Copyright 2013, Royal Society of Chemistry.

This review summarizes the recent progress in visual neuromorphic systems based on FET devices. Section 2 introduces the fundamental semiconductor materials and key FET architectures (FGFETs, FeFETs, OECTs, and EGTs), with their operating mechanisms (Figure 2). Section 3 discusses the operational principles of biological vision and the structural design of artificial visual neuromorphic systems, including visual and multisensory integration based on different FETs. Finally, an outlook on the future challenges and opportunities in this field is provided to inspire the next generation of artificial vision‐perception systems.

## 2 Classification of FETs

FETs, as representative three‐terminal devices, play an essential role in modern electronics owing to their outstanding electrical characteristics and versatility across a wide range of applications [39]. Their operation is based on the precise modulation of carrier concentration within the semiconductor channel via an external electric field, enabling controlled and efficient current regulation. Consequently, FETs are extensively employed in critical domains such as signal amplification, switching circuits, and memory elements [40].

### 2.1 Semiconductor Materials for FETs

**FIGURE 3:**
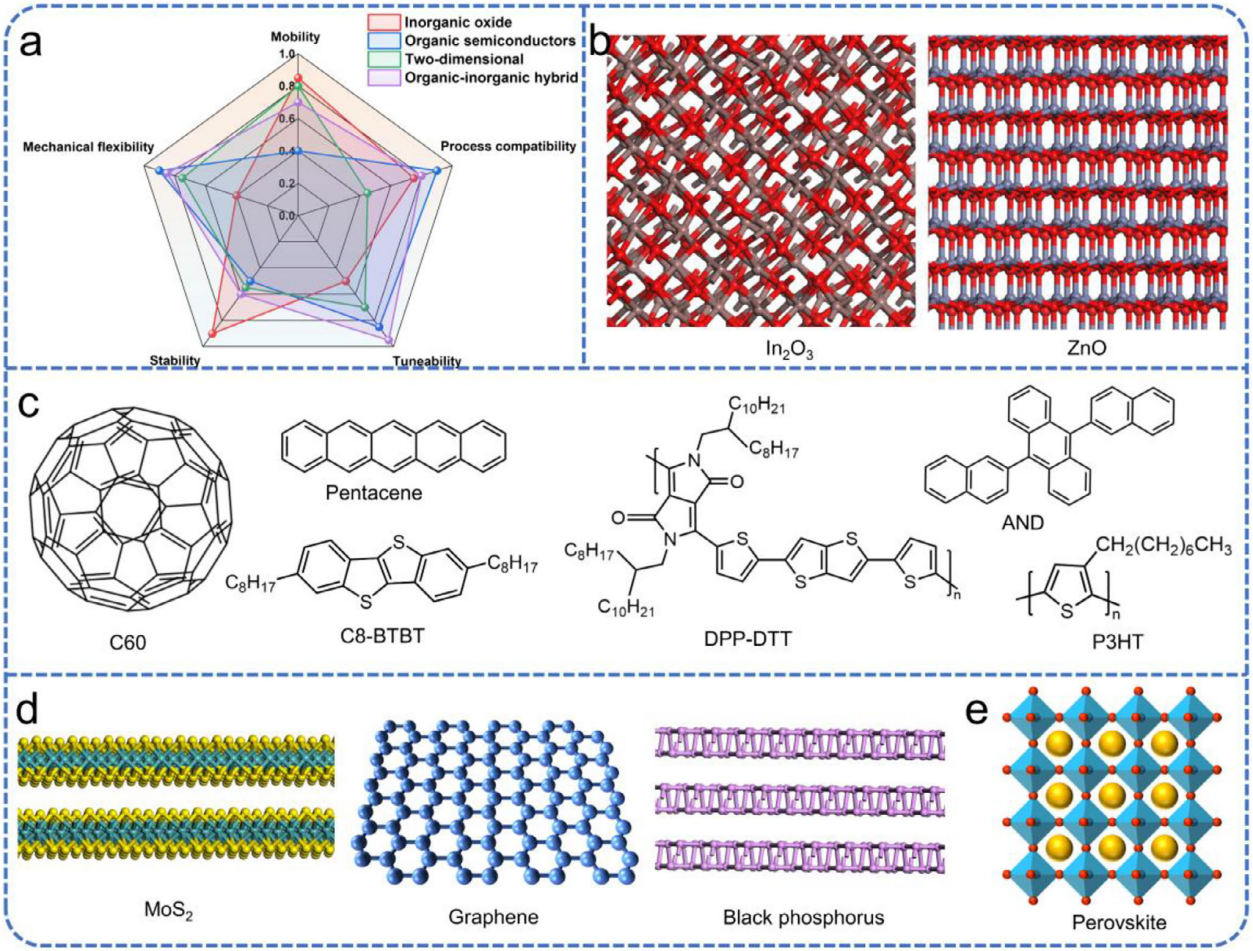
Schematic illustrations of representative semiconductor structures and molecular formulas. (a) Radar chart comparing the properties of different semiconductor materials based on normalized (0–1) performance metrics. (b) Inorganic oxide semiconductors. (c) Organic semiconductors. (d) 2D semiconductor materials. (e) Organic–inorganic hybrid perovskites.

In FETs, the carrier mobility of the semiconductor channel traditionally dictates switching speed and driving capability. However, for visual neuromorphic systems, the requirements extend beyond conventional logic performance. The channel layer must not only emulate synaptic plasticity through precise conductance modulation but also, in many cases, serve as a photoactive medium for integrated sensing and signal pre‐processing. Consequently, the selection of semiconductor materials involves a critical trade‐off between electrical performance, processing complexity, and functional versatility in mimicking the biological retina (Figure 3a) [41].

Inorganic oxide semiconductors, such as amorphous indium gallium zinc oxide (a‐IGZO), In_2_O_3_, and ZnO (Figure 3b), have transitioned from the backbone of high‐performance microelectronics to key candidates for neuromorphic vision [42, 43, 44]. These materials offer high carrier mobility and excellent batch‐to‐batch uniformity, which are essential for the large‐scale integration of synaptic arrays [45, 46, 47]. For visual applications, their inherent wide bandgap provides optical transparency, making them ideal for transparent or UV/deep ultraviolet (DUV) neuromorphic electronics. Furthermore, oxygen vacancies within these oxides can be strategically exploited as charge traps to emulate the short‐term and long‐term memory dynamics of biological synapses. Despite these advantages, their limited light absorption in the visible spectrum often necessitates the integration of additional photosensitizers to achieve the broad‐band detection required for artificial visual perception.

Organic semiconductors, including both small molecules and polymers, offer a distinct set of advantages, particularly for neuromorphic systems requiring mechanical flexibility or biological interfacing (Figure 3c) [48, 49, 50, 51, 52, 53, 54]. Their primary appeal lies in their structural tuneability and solution‐processability, which enable low‐cost, large‐area fabrication on conformable substrates [52, 55, 56, 57, 58]. From a neuromorphic perspective, their molecular structures can be chemically tailored to respond to specific light wavelengths, allowing for intrinsic sensing capabilities without the need for external photodetectors [59, 60]. Additionally, the hybrid ionic‐electronic transport mechanisms found in many organic materials closely mimic the ionic signaling prevalent in biological neural networks. However, these benefits are often tempered by lower carrier mobilities resulting from disordered molecular packing [61]. Organic devices also face significant challenges regarding environmental stability, as sensitivity to oxygen and moisture can lead to synaptic weight drift and performance degradation over time.

2D materials, such as MoS_2_, graphene, and black phosphorus (BP) (Figure 3d), have emerged as highly promising candidates due to their atomic‐scale thickness and exceptional interfacial tunability [62, 63, 64, 65, 66, 67, 68, 69, 70]. The high surface‐to‐volume ratio of 2D layers makes their conductivity extremely sensitive to external stimuli, including incident light and surface charge—a trait that is ideal for constructing high‐sensitivity artificial retinas [71]. Furthermore, their thinness effectively suppresses short‐channel effects, facilitating the ultra‐high‐density integration necessary for complex neural networks [72]. Nevertheless, the implementation of 2D materials is hindered by the technical difficulty of large‐scale, high‐quality film growth. Many 2D materials also suffer from chemical instability [73]; such as, the rapid oxidation of black phosphorus necessitates sophisticated encapsulation techniques to maintain reliable long‐term synaptic functionality in ambient conditions.

Besides, by combining the complementary advantages of individual component materials, the hybrid materials achieve high performance, tunable band structures, and multifunctional integration [74, 75, 76, 77, 78, 79, 80, 81, 82, 83]. Among these, organic‐inorganic hybrid perovskites have emerged as a transformative class of materials for neuromorphic visual systems (Figure 3e), offering a unique combination of high carrier mobility, large absorption coefficients, and tunable bandgaps [78]. These materials are particularly well‐suited for integrated sensing‐and‐processing tasks because their crystalline structure allows for efficient photon harvesting across the visible spectrum, while their soft ionic lattice facilitates controlled ion migration under electric fields. In a FET configuration, this ion migration can be exploited to emulate biological synaptic plasticity, as the movement of organic cations or halide vacancies effectively modulates the channel conductance in an analog fashion. Such dual‐functionality enables the design of one‐transistor synapses that simultaneously detect light and store visual information. However, despite their remarkable performance, hybrid perovskites face significant hurdles regarding long‐term stability and toxicity. Their sensitivity to moisture, heat, and prolonged light exposure can lead to structural degradation and instability. Furthermore, the presence of deep‐level trap states and high leakage currents—often originating from grain boundaries or interface defects—can degrade the linearity and symmetry of weight updates, posing a challenge for the reliable implementation of large‐scale, high‐accuracy visual neuromorphic arrays.

### 2.2 Dielectric Materials for FETs

Meanwhile, the choice of dielectric layer can fundamentally alter the operating mechanisms of transistors, thereby significantly influencing their performance and response characteristics. This section systematically reviews the operating principles of FGFETs, FeFETs, OECTs, and EGTs.

#### 2.2.1 Floating‐Gate Field‐Effect Transistors (FGEFTs)

In FGFETs, a floating gate is embedded within a dielectric layer between the control gate and the semiconductor channel, enabling non‐volatile charge storage [84, 85]. When a gate bias is applied, carriers can be injected into the floating gate via thermal excitation or quantum tunneling. Upon removal of the gate voltage, the charges become trapped within the floating gate, and the stored charges create an electric field that screens the gate potential, leading to a shift in the threshold voltage and thereby modulating channel conductivity. By applying sequential gate voltage pulses, the quantity of trapped charge can be precisely controlled, allowing the device to emulate synaptic weight updates through analog conductivity tuning [86]. This ability to progressively alter trapped charge with pulse number makes FGFETs particularly suitable for implementing synaptic plasticity in neuromorphic devices.

**FIGURE 4:**
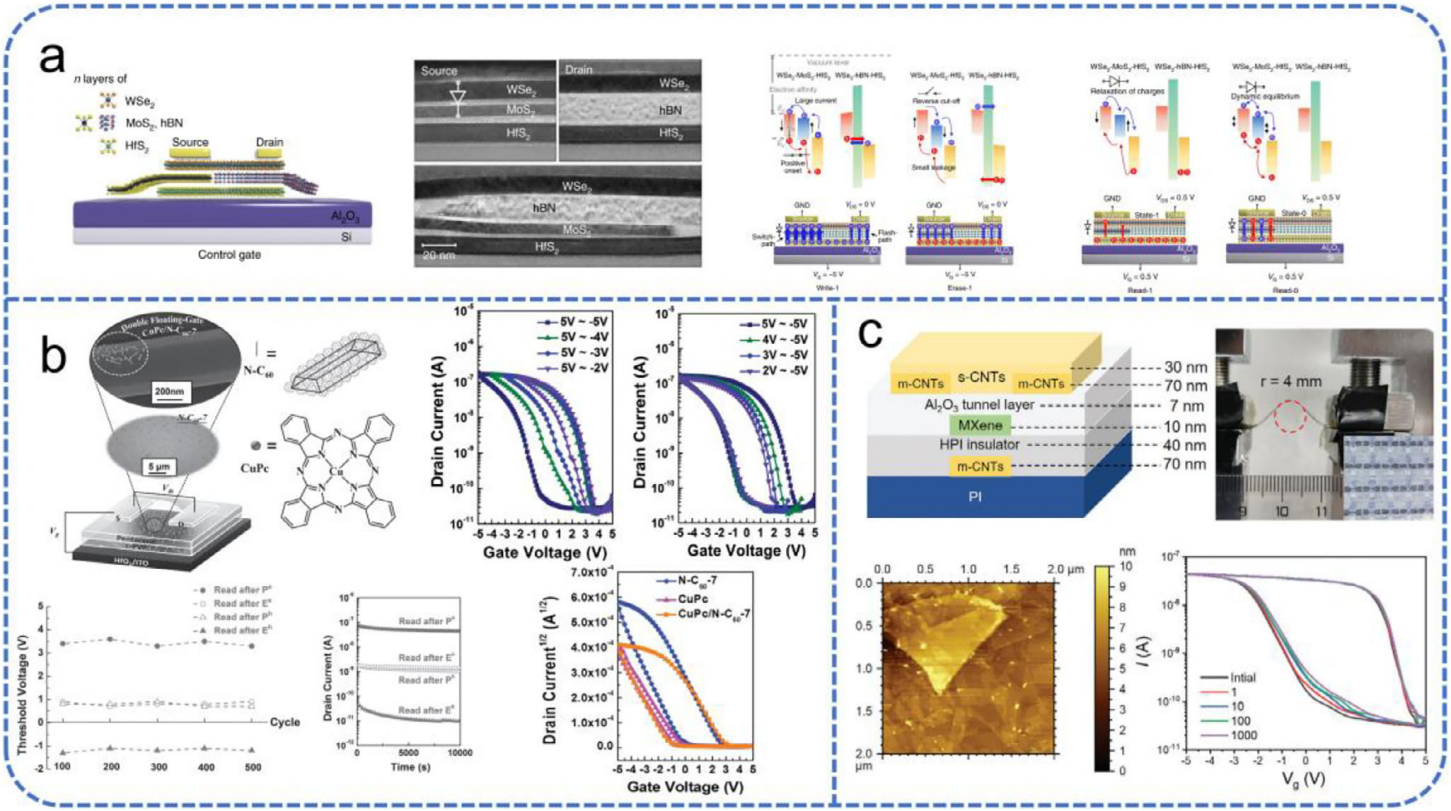
Schematic overview of different FGFET device architectures. (a) Schematic structure and mechanisms of 2D SFG memory. Reproduced with permission [87]. Copyright 2018, Springer Nature. (b) Schematic cross‐section, optical microscopy, TEM images, and transfer characteristics of a pentacene‐based FGFET memory. Reproduced with permission [89]. Copyright 2014, Wiley. (c) Schematic diagram of MXene‐based FGFET device, AFM image, and transfer characteristic following different bending cycles (4 mm). Reproduced with permission [90]. Copyright 2024, Wiley.

Recent advances have leveraged novel materials and heterostructures to enhance FGFET performance. Liu et al. reported a van der Waals (vdW) heterostructure‐based semi‐floating‐gate device, as illustrated in Figure 4a [87]. By exploiting the unique band structures of 2D materials and the lattice‐mismatch‐insensitive nature of vdW interfaces, this design achieves rapid charge storage and release. It combines write speed and extended refresh intervals, significantly improving overall memory performance while reducing the power overhead associated with frequent refresh operations. Meanwhile, double‐layer FGFETs can effectively adjust the charge‐trapping [88, 89]. Chang et al. implemented a dual floating‐gate structure comprising single‐crystal C_60_ needles and CuPc nanoparticles to achieve non‐volatile memory functionality in low‐voltage FETs (Figure 4b) [89]. The high surface area of C_60_ needles, combined with the uniform distribution of CuPc nanoparticles, enhances charge trapping and storage efficiency, allowing stable memory operation at low voltages. This work demonstrates how the integration of complementary charge‐trapping materials can significantly improve FET performance and energy efficiency.

Beyond rigid architectures, the development of flexible FGFETs has become increasingly critical for enabling future embodied intelligence and wearable electronics. Zhu et al. reported a flexible FGFET employing Ti_3_C_2_F MXene as the floating gate (Figure 4c) [90]. The device leverages MXene's high conductivity and mechanical flexibility to achieve stable synaptic responses at low operating voltages. The transistor exhibits key synaptic functionalities, which provide a novel patterning and integration strategy for MXene‐based flexible neuromorphic devices. Li et al. proposed a flexible optoelectronic synaptic transistor based on high‐quality lead‐free Cs_3_Bi_2_I_9_ nanocrystals [91]. To address retention and efficiency, Yan et al. developed a non‐volatile memory using ZnSe@ZnS core–shell quantum dots (QDs) as the tunneling dielectric, achieving long charge retention and high tunneling efficiency [92]. Additionally, graphene QDs and multilayer graphene (MLG) have been widely adopted as floating‐gate materials [93].

Thus, by optimizing floating‐gate materials, dielectric engineering, and device architecture, the charge‐trapping characteristics of FGFETs can be precisely tailored. These advances significantly enhance device performance in terms of memory window, stability, non‐volatility, and robustness, making FGFETs a versatile platform for next‐generation memory and neuromorphic computing applications.

#### 2.2.2 Ferroelectric Field‐Effect Transistors (FeFETs)

FeFETs represent a significant departure from conventional charge‐trapping devices by integrating ferroelectric materials as the gate dielectric to achieve non‐volatile functionality. The core operating principle relies on the field‐induced switching of the ferroelectric layer's spontaneous polarization (P) [94, 95]. When a gate voltage (Vg​) is applied, the alignment of ferroelectric domains induces a persistent remnant polarization that modulates the carrier concentration in the underlying semiconductor channel through the field effect. This polarization remains stable upon removal of the external field, allowing FeFETs to maintain distinct “on” and “off” states with ultra‐low power consumption. Thus, FeFETs are widely explored for applications in nonvolatile memory, energy‐efficient sensors, artificial synapses, and neuromorphic computing.

**FIGURE 5:**
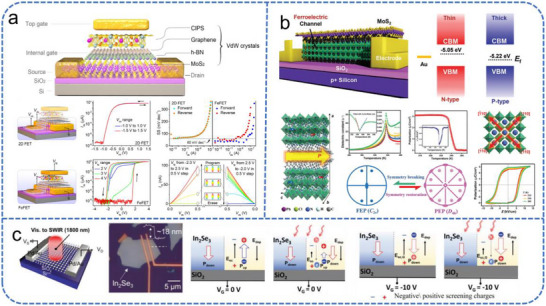
Schematic overview of different FeFET device architectures. (a) Schematic diagram of a MoS_2_/h‐BN/graphene/CIPS vdW FeFET and the electric transport properties. Reproduced with permission [96]. Copyright 2021, Springer Nature. (b) Characterization of 2D‐PVK‐based FeFETs: from device schematic and heterojunction band to the crystal structure and ferroelectricity of the material. Reproduced with permission [2]. Copyright 2024, Wiley. (c) Schematic diagram of the α−In_2_Se_3_ device, an optical micrograph, and the gate‐modulated photo‐induced polarization switching mechanism. Reproduced with permission [97]. Copyright 2023, Wiley.

To overcome the challenges of interface degradation and depolarization fields inherent in bulk ferroelectrics, recent research has pivoted toward vdW engineering and low‐dimensional heterostructures. Wang et al. developed a vdW‐assembled FeFET utilizing a CuInP_2_S_6_ ferroelectric layer integrated with a bipolar graphene interlayer (Figure 5a) [96]. The vdW interface minimizes atomic diffusion and dangling bonds, while the graphene layer effectively compensates for the ferroelectric polarization, suppressing the detrimental depolarization field. The device demonstrates robust performance with a data retention time exceeding 10 years and endurance over 10^4^ cycles, confirming its reliability for practical applications. Taking a different approach to interface engineering, Xu et al. constructed a heterojunction between a 2D Ruddlesden–Popper hybrid perovskite (2D‐PVK) and MoS_2_ (Figure 5b) [2]. Unlike conventional ferroelectric gate insulators, the 2D‐PVK in this architecture directly modulates channel conductivity and exhibits unique charge‐polarity switching (from n‐ to p‐type behavior). The device delivers record‐high performance metrics, including a large hysteresis window of ≈177 V, a high on/off current ratio >10^5^, fast programming speed, and long‐term retention. This work highlights the potential of heterojunction engineering based on band alignment and charge transfer for developing high‐performance nonvolatile memories.

The convergence of ferroelectricity with light‐sensitive semiconductors has further positioned FeFETs as a foundational technology for visual neuromorphic systems [97, 98]. Unlike traditional image sensors that require separate processing units, FeFETs can simultaneously detect, store, and process optical information. A prime example is the multifunctional platform reported by Li et al., which utilizes wrinkle‐free 2D α−In_2_Se_3_​ to integrate photodetection, reconfigurable logic, and light‐processing capabilities (Figure 5c) [97]. Due to the intrinsic semiconductor‐ferroelectric nature of α−In_2_Se_3_, these transistors exhibit a high current‐switching ratio (>10^6^) and a strong photoresponse across visible to short‐wave infrared wavelengths. This “all‐in‐one” capability allows for the emulation of retinal functions where preprocessing occurs directly at the sensing node.

Expanding on this complexity, Luo et al. demonstrated a dual‐gate 2D FeFET with coupled ferroelectric polarization [98]. This configuration allows independent carrier concentration tuning via each gate, yielding multiple conductive states suitable for complex logic operations and synaptic emulation. The versatility of this platform is further supported by the diverse library of ferroelectric materials currently under investigation, including inorganic oxides like BaMgF_4_, LiNbO_3_ [99, 100, 101], BaTiO_3_ [102, 103], ZrO_2_ [104], and HfO_2_ [105, 106, 107], as well as flexible organic polymers such as P(VDF−TrFE) [108, 109]. Together, these advancements in material synthesis and device architecture underscore the potential of FeFETs to serve as the backbone for next‐generation intelligent perception systems.

#### 2.2.3 Organic Electrochemical Field‐Effect Transistors (OECTs)

OECTs are devices that modulate the conductivity of a conductive channel through electrochemical reactions. Their operation relies on the electrochemical doping and de‐doping of organic semiconductors, driven by ion migration within an electrolyte under an applied gate voltage. OECTs uniquely combine ion doping and redox mechanisms, enabling prolonged stable channel conductivity, which makes them suitable for non‐volatile memory, bio‐inspired computing, and multisensory integration [110]. Driven by a gate voltage (Vg), ions from an electrolyte are injected into the bulk of the organic film, leading to a persistent change in conductivity. This volumetric response allows OECTs to achieve exceptionally high transconductance (g_m_) and stable multi‐state conductivities at sub‐1 V operating voltages, making them ideal for energy‐efficient synaptic emulation and multisensory integration in flexible or bio‐integrated platforms [111].

**FIGURE 6:**
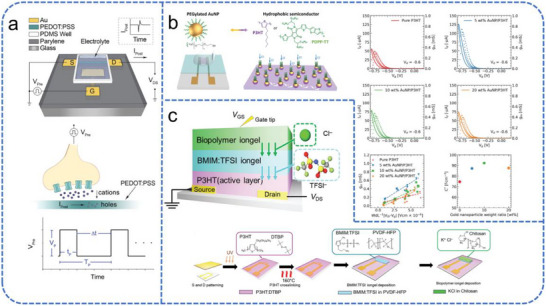
Schematic overview of different OECT device architectures. (a) A typical OECT device structure and writing diagram. Reproduced with permission [112]. Copyright 2015, Wiley. (b) Illustration of the mechanism and performance characterization of hydrophobic semiconducting polymers incorporated with PEGylated AuNPs. Reproduced with permission [113]. Copyright 2024, Wiley. (c) Schematic illustration of the fabrication process and device structure of cross‐linked P3HT OECTs incorporating BMIM:TFSI ion gel and biopolymer‐based ion gel. Reproduced with permission [114]. Copyright 2024, Wiley.

The foundational mechanism of OECT‐based neuromorphic devices was demonstrated by Gkoupidenis et al. using a poly(3,4‐ethylenedioxythiophene):poly(styrene sulfonate) (PEDOT:PSS) channel (Figure 6a) [112]. In this system, the application of a positive gate bias drives cations from the electrolyte into the PEDOT:PSS layer, effectively de‐doping the channel and decreasing its conductivity. The slow diffusion and trapping of these ions mimic the temporal dynamics of biological synapses, such as STP and LTP.

To further advance the performance and functional versatility of OECTs, recent research has focused on overcoming the intrinsic ion‐transport limitations of hydrophobic semiconductors within device architectures. Ho et al. enhanced OECT performance without modifying the intrinsic properties of hydrophobic polymer semiconductors by incorporating poly(ethylene glycol) (PEG)‐coated gold nanoparticles (AuNPs) [113], as shown in Figure 6b. The PEG layer creates a hydrophilic interface between the hydrophobic semiconductor (e.g., P3HT) and the electrolyte, facilitating ion penetration and transport. Additionally, the conductive AuNPs form percolation networks within the polymer matrix, boosting electron mobility (µ), transconductance (g_m_), and charge mobility (µC^*^). This strategy enhances the device's sensitivity and switching speed without altering the intrinsic properties of the polymer. Similarly, Lee et al. developed a novel crosslinking strategy using di‐tert‐butyl‐peroxide (DTBP) to crosslink poly(3‐hexylthiophene‐2,5‐diyl) (P3HT) and fabricated high‐performance OECTs with dual‐ion gels [114], as depicted in Figure 6c. The crosslinked P3HT was integrated with an ion gel composed of BMIM:TFSI, PVDF‐HFP, and a hydrophilic chitosan‐based polymer gel containing K^+^ and Cl^−^ ions. The resulting devices exhibited excellent electrical characteristics and synaptic behaviors, including high charge mobility and stable LTP. This architecture, combining hydrophilic ion gels with hydrophobic semiconductors, enables efficient ion‐electron mixed conduction and stable charge modulation.

In the context of visual neuromorphic systems, OECTs offer a distinct advantage: their biocompatibility and mechanical flexibility allow them to be seamlessly integrated with organic photodetectors or even directly interfaced with biological tissues [115]. This enables artificial retina applications where optical signals are converted into ionic fluxes, mimicking the signal transduction of natural photoreceptors. While OECTs currently face challenges regarding response speeds and environmental sensitivity (e.g., moisture and oxygen), emerging strategies such as vertical channel architectures, nano‐patterning, and advanced encapsulation are rapidly closing the performance gap [116]. These developments position OECTs as a versatile candidate for the next generation of low‐power, flexible neuromorphic perception systems.

#### 2.2.4 Electrolyte‐Gated Field‐Effect Transistors (EGTs)

EGTs represent a class of devices that achieve exceptionally high coupling between ionic and electronic signals, primarily through the formation of an Electric Double Layer (EDL) at the electrolyte/semiconductor interface. Under an applied gate bias, ions within the electrolyte redistribute to form a nanometer‐scale capacitor (EDL), which induces a high density of charge carriers in the semiconductor channel at remarkably low operating voltages (typically<1 V) [117]. This high‐capacitance coupling, combined with the inherent biocompatibility and mechanical flexibility of electrolytes, makes EGTs particularly suitable for low‐power neuromorphic hardware and bio‐inspired visual systems.

**FIGURE 7:**
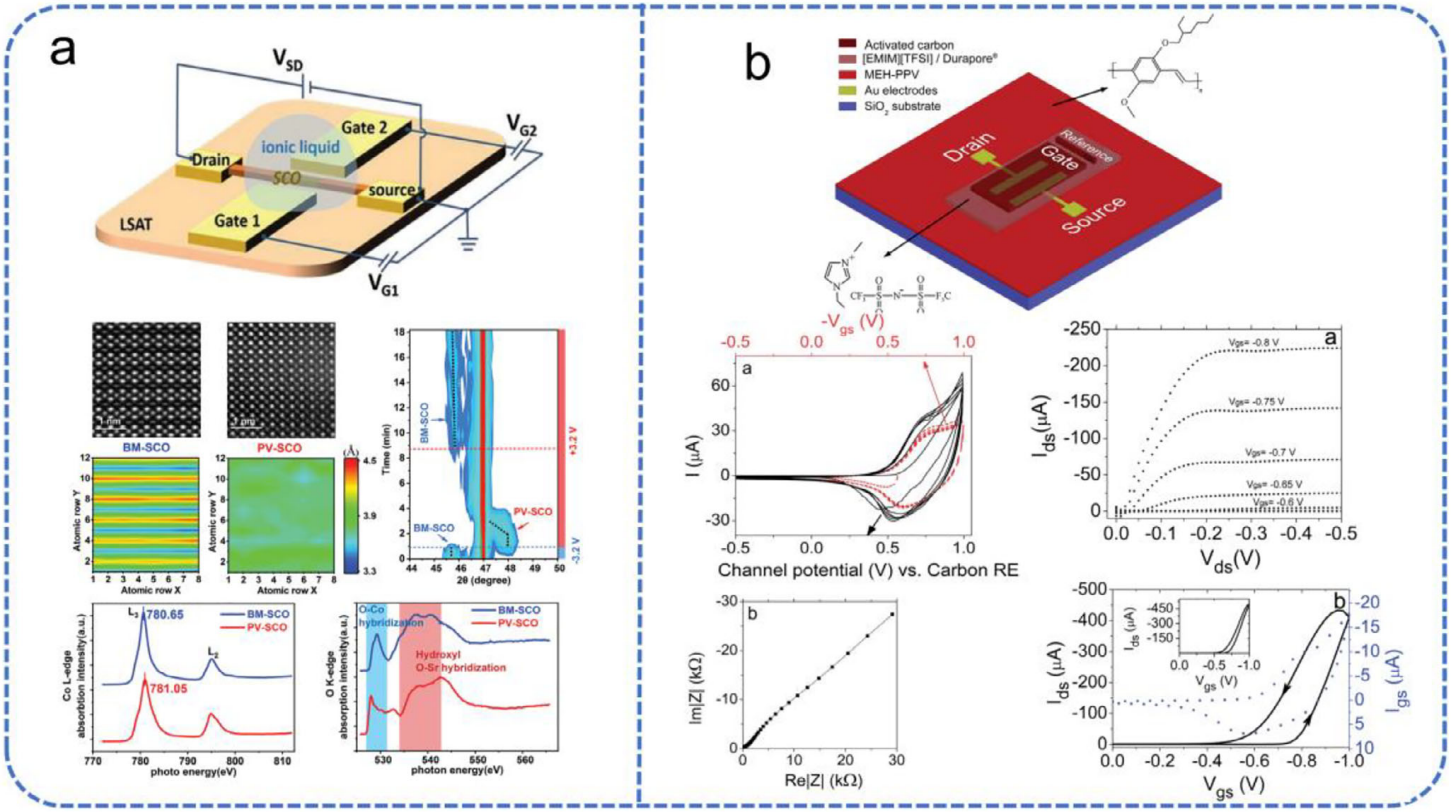
Schematic overview of different EGT device architectures. (a) Schematic of a three‐terminal ionic‐liquid‐gated SCO transistor, showing the structure and the gating‐manipulated phase transformation between BM‐SCO and PV‐SCO phases. Reproduced with permission [118]. Copyright 2019, Wiley. (b) Device structure and electrical characteristics of an [EMIM][TFSI]‐gated MEH‐PPV transistor with an activated carbon gate electrode. Reproduced with permission [120]. Copyright 2014, Royal Society of Chemistry.

Huang et al. developed an EGT synaptic device using SrCoO_x_ (SCO) thin films to overcome the limited retention of conventional proton‐based synaptic transistors (Figure 7a) [118]. Employing the ionic liquid DEME‐TFSI as the gate medium, the device controls the reversible insertion and extraction of oxygen ions in the SCO film via electrolyte gating, and this device achieves prolonged information retention and exhibits enhanced stability in synaptic simulations. Meanwhile, Li et al. reported a reconfigurable EGT with a BaSnO_3_ channel that utilizes EDL and ion migration mechanisms for multimodal sensing [119]. These works exploit the dynamic ion modulation in EGTs to emulate synaptic plasticity within a three‐terminal architecture, integrating memory and processing functions to overcome von Neumann bottleneck limitations and provide a low‐power, compact hardware solution for neuromorphic computing.

The structural simplicity of EGTs also allows for innovative gate engineering to reduce system complexity. Sayago et al. demonstrated a low‐voltage EGT utilizing a high‐surface‐area activated carbon gate electrode and an [EMIM][TFSI] ionic liquid (Figure 7b) [120]. The porous nature of the carbon electrode enhances charge storage via the EDL effect, enabling precise modulation of the MEH−PPV polymer channel at sub‐1 V levels without requiring a separate reference electrode. These advancements, along with the exploration of diverse solid‐state and gel electrolytes [121, 122], have expanded the operational environment of EGTs, making them highly adaptable for visual neuromorphic systems that require low‐power monitoring. By mimicking the ionic flux of biological retinal synapses, EGTs can directly integrate with photosensitive layers to perform complex spatiotemporal visual processing at the sensing node.

#### 2.2.5 Comparative Overview of Transistor Architectures

To provide a comparative perspective, the key performance metrics of the aforementioned transistor architectures—including dielectric materials, carrier mobility, on/off ratio, and operating voltage—are summarized in Table 1.

**TABLE 1:** A summary of dielectric layer materials, carrier mobilities, on/off ratios, and operating voltages of different FET devices.

Category	Dielectric layer materials	Carrier mobility (cm^2^ V^−1^ s^−1^)	ON/OFF ratio	Operating voltage	Refs.
FGFET	P(VDF‐TrFE‐CFE)	>0.2	>10^2^	±50 V	[123]
	Al_2_O_3_	0.1	3 × 10^4^	±5 V	[88]
	SiO_2_/PS	0.6	>10^5^	±80 V	[124]
	h‐BN	15	/	±40 V	[125]
FeFET	SiO_2_/HfO_2_	312 488	>10^8^	50 V/5 V	[94]
	(3‐Pyrrolinium)(CdCl_3_)	1.28 ± 0.41	>10^5^	±100 V	[126]
	PVDF‐TrFE	/	>10^3^	±80 V	[127]
OECT	NaCl	7 × 10^4^	> 6 × 10^3^	0.6 V	[128]
	PIL ionogel	5.7	1.2 × 10^5^	−0.5–1.0 V	[129]
	NaClO_4_	/	10^5^	/	[130]
EGT	α6T/PBS	4 × 10^−^^2^	10^2^∼10^3^	−50 mV∼−1 V	[131]
	PS‐PMMA‐PS/[EMIM][TFSI]	2.4	2.15 × 10^5^	<2 V	[132]
	PVDF/[EMI][TFSI]	2.06/2.81	>10^4^/10^5^	< 2 V/< 1 V	[133]

Generally, most FGFETs and FeFETs are characterized by high carrier mobilities and large on/off current ratios, making them excellent for high‐speed logic and robust non‐volatile memory. However, their operation typically requires higher voltages to overcome the intrinsic energy barriers of solid‐state dielectrics or to facilitate quantum tunneling. In contrast, OECTs and EGTs operate at much lower voltages (typically around 1 V or lower) and consume less energy. Their functionality relies on ionic migration and interfacial electrochemical processes rather than electrostatic induction through a thick dielectric bulk. While OECTs and EGTs often exhibit lower electronic mobilities due to the presence of ions and organic materials, their ability to mimic the ionic nature of biological systems makes them uniquely qualified for the next generation of flexible, wearable, and bio‐integrated neuromorphic visual systems.

## 3 Neuromorphic Visual Perception Functions and Applications

### 3.1 Biological Visual System Heuristics

**FIGURE 8:**
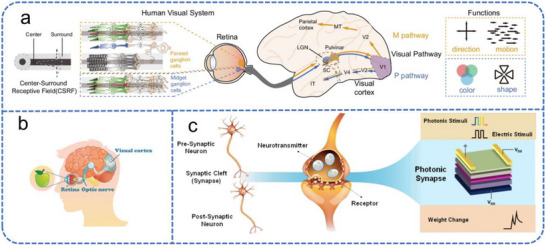
Human visual pathways and neural synapse emulation. (a) Human visual pathways from the retina to the cortex and their functions. Reproduced with permission [138]. Copyright 2024, Spring Nature. (b) Biological visual system. Reproduced with permission [153]. Copyright 2021, Wiley. (c) Comparison of the functions and structures of biological synapses and artificial synapses. Reproduced with permission [150]. Copyright 2024, Springer Nature.

In the human brain, 80 % of external information is perceived through the visual system [134]. Biological and bioinspired visual pathways are illustrated in Figure 8. The visual system is a sophisticated biological architecture that integrates multiple organs and neural circuits to transform light into coherent perception. The core components of the biological pathway include the eyes, visual pathways, and the visual cortex.

Visual processing begins when light enters the eye and is focused by the cornea and lens onto the retina. Here, specialized photoreceptor cells convert light stimuli into electrochemical signals. These photoreceptors are functionally divided into two types: rods and cones [6, 135, 136]. Rod cells are highly sensitive to dim light and mediate scotopic (low‐light) vision, whereas cone cells are responsible for photopic (bright‐light) vision and color perception. The electrical signals generated by photoreceptors are relayed via bipolar cells to retinal ganglion cells. The axons of these cells form the optic nerve, which carries the information to the lateral geniculate nucleus (LGN) of the thalamus for initial processing.

Subsequently, preprocessed visual data is projected to the primary visual cortex (V1) in the occipital lobe. V1 is fundamental for extracting basic visual features such as edges, orientations, and spatial frequencies. This information is then integrated in the secondary visual cortex (V2) to discern shapes and spatial structures. For advanced processing, the data stream diverges: information concerning color and form is forwarded to the extrastriate cortex (V4) for detailed analysis, while motion‐related signals are routed via the dorsal stream to the middle temporal cortex (MT) for perceiving direction and velocity, enabling dynamic tracking and spatial localization [137, 138]. This intricate, hierarchical collaboration allows for clear environmental perception, facilitating advanced functions like color recognition, dynamic range adjustment, and depth perception.

At the synaptic level within biological neural networks, external stimuli trigger action potentials at the presynaptic membrane. This leads to the release of neurotransmitters, which cross the synaptic cleft and bind to receptors on the postsynaptic membrane, thereby modulating the postsynaptic current. When the presynaptic stimulus ceases, neurotransmitters are gradually cleared, causing the postsynaptic current to decay slowly to its baseline state [139, 140]. This dynamic modulation is crucial for the synaptic storage, processing, and transmission of information [141, 142].

Guided by these biological principles, the following section explores how FET‐based devices can be engineered to emulate key functions of the human visual perception system, paving the way for efficient visual perception applications.

### 3.2 Design Principles of FET‐Based Visual Neuromorphic Systems

Research into visual neuromorphic systems aims to replicate the biological efficiency of the human visual system by integrating sensing and processing into a unified hardware framework [143, 144, 145, 146]. Within this domain, FET‐based architectures have emerged as a premier platform due to their structural versatility and the precise controllability of their electronic states. These devices facilitate the co‐location of sensing, memory, and computation—a paradigm that is essential for realizing event‐driven processing and high‐fidelity multimodal integration [147].

In a typical FET‐based visual system, photosensitive components integrated within the channel, dielectric, or gate stack serve as the primary transducers. These materials directly detect environmental variations in light intensity, spectral composition (color), and spatial orientation [148]. Upon photon absorption, generated carriers modulate the channel conductance, effectively converting optical stimuli into internal synaptic weight updates. This direct conversion mechanism enables the device to perform in situ signal integration, where the transistor only generates a significant output or fires when a salient stimulus exceeds a specific threshold. This behavior mimics the sparse, event‐driven encoding of biological neurons, significantly reducing redundant data transmission and power consumption.

The functional diversity of FETs—ranging from floating‐gate charge trapping and ferroelectric polarization to ionic/electrolyte gating—provides a rich toolkit for emulating the complex plasticity of the visual pathway. Each mechanism offers unique temporal dynamics, such as STP for motion detection and LTP for pattern recognition. By leveraging these tunable properties, FET arrays can be configured to emulate the hierarchical processing stages of the biological system, from the initial retinal preprocessing to the sophisticated feature extraction occurring in the visual cortex. This framework establishes FETs as a versatile hardware foundation for low‐power, intelligent visual systems capable of real‐time perception and adaptive learning. The following section provides a detailed overview of the various neuromorphic device architectures developed within this framework.

#### 3.2.1 FGFETs for Visual Neuromorphic Systems

While the fundamental physics of charge trapping in FGFETs were discussed in Section 2.2, recent research has pivoted toward utilizing these mechanisms for sophisticated visual perception tasks, ranging from retinal edge enhancement to multispectral recognition. By engineering heterojunctions and incorporating photo‐active molecular layers, FGFETs can achieve the sensing‐memory‐processing synergy required for advanced neuromorphic vision.

**FIGURE 9:**
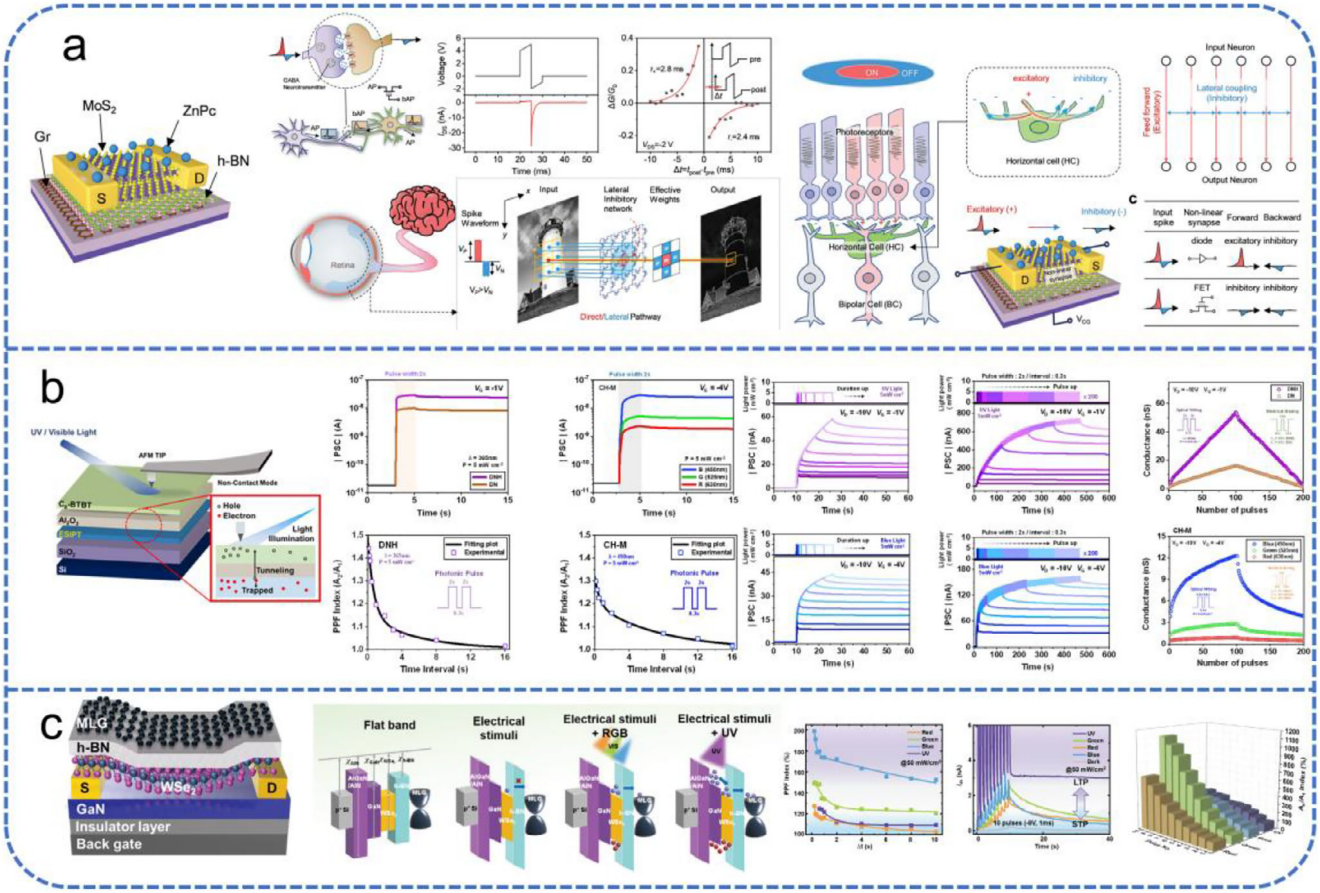
Schematic illustration of FGFET‐based devices for neuromorphic visual perception. (a) Structure of a FGFET with bidirectional rectification for emulating retinal lateral inhibition and edge‐enhanced image processing. Reproduced with permission [149]. Copyright 2022, Royal Society of Chemistry. (b) Structure, characterization, and modulation of photonic synaptic transistors. Key metrics include: molecular PSC/PPF comparison, light‐pulse‐dependent PSC response, and LTP/LTD behavior under combined optical/electrical modulation. Reproduced with permission [150]. Copyright 2024, Springer Nature. (c) Device structure, energy band diagrams under different operational states, and PPF/SNDP indices of the photonic synaptic transistor under visible and UV illumination. Reproduced with permission [151]. Copyright 2024, Wiley.

One of the most critical functions of the biological retina is spatial feature extraction through lateral inhibition. Hu et al. emulated this behavior using a 2D FGFET based on a MoS_2_/h−BN/graphene heterostructure (Figure 9a) [149]. The nonlinear conductivity of the MoS_2_ channel enables bidirectional yet selectively inhibitory current transmission. This configuration establishes an on‐center/off‐surround receptive field, effectively enhancing image edge features. The work provides an innovative strategy for developing low‐power, tunable neuromorphic vision devices. While promising, the transition of such 2D heterostructure devices into large‐scale arrays remains contingent on improving fabrication uniformity and mitigating signal crosstalk during parallel weight updates. Beyond spatial processing, FGFETs have demonstrated remarkable potential in chromatic and multispectral discrimination. Jeong et al. integrated a photo‐responsive organic molecule, CH‐M, as the floating‐gate material to achieve high‐fidelity RGB color differentiation (Figure 9b) [150]. Upon photoexcitation, proton transfer within the CH‑M molecule alters its dipole moment, modulating the charge distribution between the floating gate and the channel layer and thereby changing the postsynaptic current. This light‑induced dipole effect significantly enhances the device's photosensitivity and enables wavelength‑dependent photoresponses, facilitating effective RGB color discrimination and demonstrating its promise for efficient visual perception.

Su et al. developed a vertically stacked 2D/3D semiconductor heterostructure to create a dual‐channel FGFET with a broad spectral response extending from the visible to the UV range (Figure 9c) [151]. Combined with electrical and optical stimulation, the device effectively emulates fundamental synaptic behaviors, providing an innovative architecture for high‐performance tetrachromatic vision systems. After network training, the device achieved a 96.6 % recognition accuracy for UV images, underscoring its potential for high‐performance machine vision.

Meanwhile, the reconfigurability of FGFETs allows for the emulation of complex neural circuits within the retina and visual cortex. Peng et al. designed a split FGFET using 2D WSe_2_, which exhibits reconfigurable positive/negative photoresponse and tunable conductivity [138]. This split‐gate device emulates neural circuits of the retina and visual cortex, which achieves reconfigurable photoresponse and synaptic weight modulation via photovoltage gating, opening a new avenue for the seamless integration of complex visual perception and cognitive functions in neuromorphic vision chips. Despite these significant device‐level breakthroughs, the next frontier for FGFET‐based vision lies in system‐level validation. Future research must prioritize high‐density integration and the development of peripheral circuitry to fully transition from individual synaptic emulators to comprehensive, in‐sensor neuromorphic vision chips.

#### 3.2.2 FeFETs for Visual Neuromorphic Systems

Research into FeFET‐based visual neuromorphic systems has advanced significantly, driven by the unique ability of these devices to merge the non‐volatile storage of ferroelectric materials with the photoresponsive nature of semiconductor channels. This synergy enables the monolithic integration of sensing, memory, and computation—a critical requirement for efficient neuromorphic vision.

**FIGURE 10:**
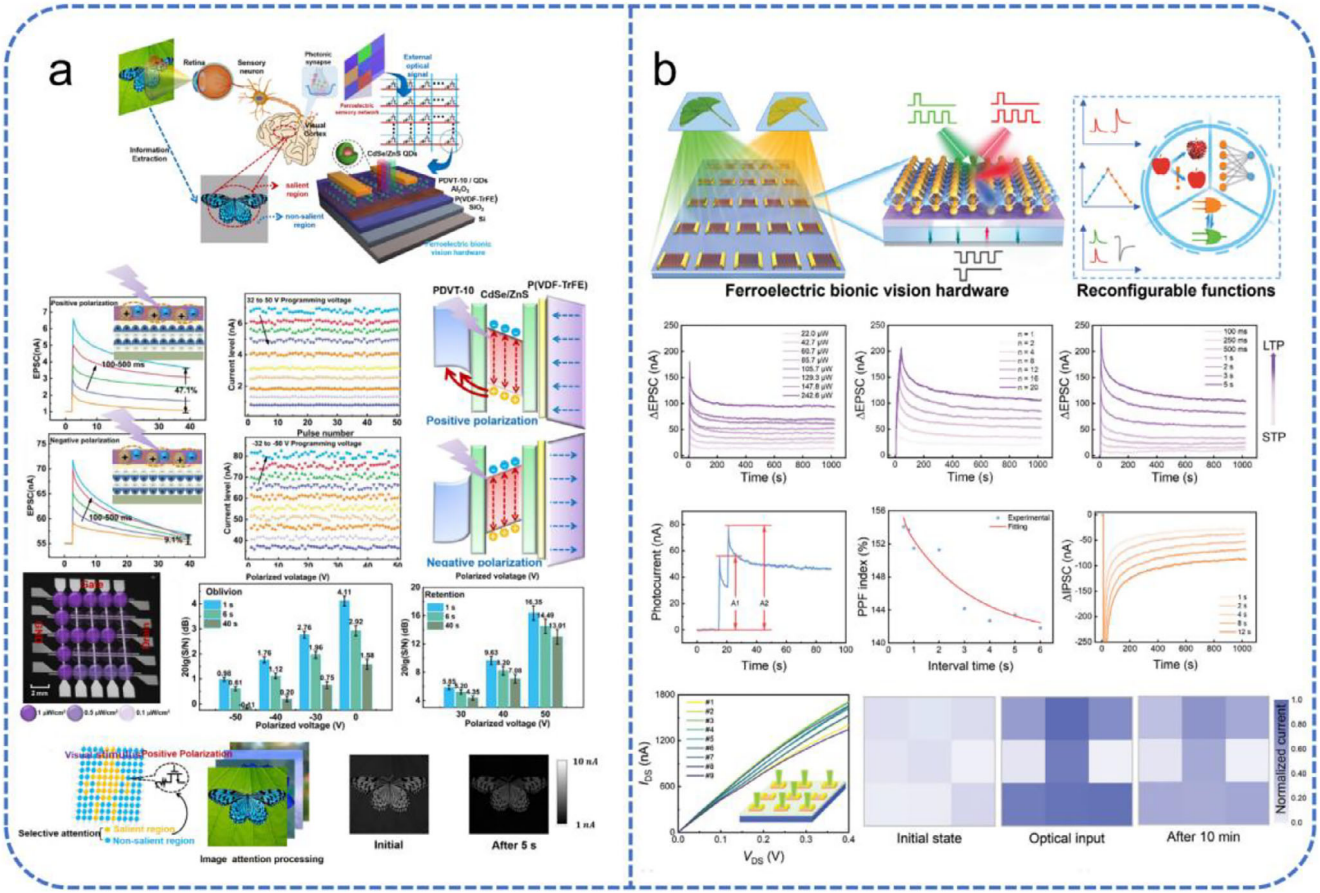
Schematic illustration of FeFET‐based devices for neuromorphic visual perception. (a) Schematic comparison of the biological visual perception system and an artificial ferroelectric biomimetic vision system, illustrating selective attention via polarization, time‐varying signal‐to‐noise ratio under positive/negative polarization, and image attention processing. Reproduced with permission [152]. Copyright 2022, Springer Nature. (b) Schematic of the 2D ferroelectric optoelectronic transistor platform and its feature modulation mechanism, emulating synaptic characteristics and normalized current distribution under high‐intensity learning. Reproduced with permission [101]. Copyright 2024, Wiley.

Recent breakthroughs have demonstrated FeFETs capable of sophisticated color and dynamic information processing. For instance, Liu et al. developed a FeFET system that distinguishes light wavelengths to process chromatic data [6]. Building on this, Yu et al. implemented a programmable ferroelectric biomimetic visual hardware (FeBVH) designed to simulate the selective attention mechanism of the human visual system (Figure 10a) [152]. By leveraging the polarization states of the ferroelectric layer, the FeBVH achieves a tunable photoresponse. Under positive polarization, the device efficiently extracts short‐wavelength signals; this linear drain current and wavelength dependence significantly enhance image classification. Consequently, neural network recognition accuracy improved from 69.7 % to 95.7 %, showing the potential of FeBVH for autonomous driving and intelligent monitoring.

The integration of multi‐modal functions has been further realized through heterostructure FeFETs. As illustrated in Figure 10b, these devices can simultaneously perform reconfigurable sensing, memory, and logic operations [101]. By utilizing the synergistic interaction of optical and electrical stimuli, these heterostructures emulate the transition from STP to LTP. This mimics the biological transition from short‐term memory (STM) to long‐term memory (LTM), allowing the system to learn and retain image data based on varying optical intensities. Additionally, further research has expanded these capabilities to include retinal color recognition [153] and high‐accuracy recognition driven by light‐induced polarization effects [154].

Despite these milestones, several hurdles remain for the practical deployment of large‐scale FeFET arrays. Frequent write operations often lead to synaptic weight‐update fatigue and degraded retention [155]. Furthermore, material incompatibility between the photoactive semiconductors and ferroelectric components complicates the fabrication of high‐density arrays. Future research must address these stability and integration challenges to transition from individual device demonstrations to fully integrated, large‐scale artificial visual systems.

#### 3.2.3 OECTs for Visual Perception Applications

OECTs offer a distinct bio‐inspired approach to neuromorphic vision by utilizing ionic modulation to process information. Unlike traditional solid‐state devices, OECTs operate through the interaction of ions and electrons, closely mimicking the electrochemical signaling found in biological synapses.

The integration of photoactive materials allows OECTs to convert optical stimuli directly into ionic currents. Chen et al. reported an organic optoelectronic synapse based on photon‐modulated electrochemical doping [156]. This device employs a donor–acceptor heterojunction of P3HT and [6, 6]‐phenyl‐C_61_‐butyric acid methyl ester (PCBM) as the photoactive layer, which exhibits excellent charge separation properties. Upon illumination, photocarriers disrupt the electrochemical doping balance, triggering compensatory ion migration from the electrolyte into the channel. By constructing a 4 × 5 synaptic array, the researchers demonstrated that each unit could independently generate light‐induced photocurrents with non‐volatile memory, enabling robust image perception and memorization at the array level. Building on this capability, a larger simulated synaptic array was further employed to realize facial recognition functionality.

**FIGURE 11:**
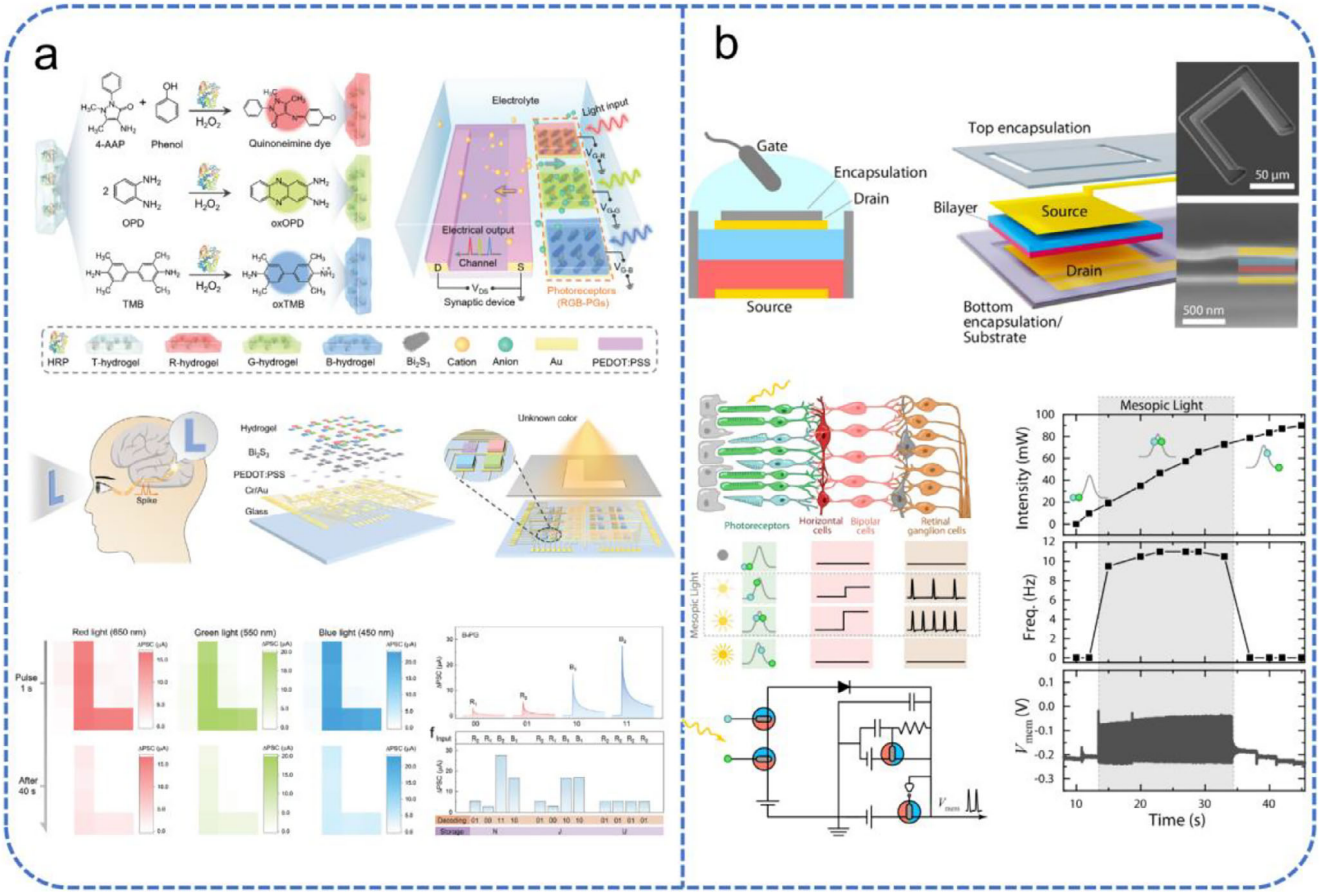
Schematic illustration of OECT‐based devices for neuromorphic visual perception. (a) An Integrated retinomorphic organic photoelectrochemical transistor system for color perception: schematic illustration comprising RGB hydrogels (color‐modulated via HRP‐catalyzed reactions with H_2_O_2_), a polymeric channel, and synaptic arrays, enabling color image sensing and memory. Reproduced with permission [157]. Copyright 2024, Wiley. (b) Structure, characterization, and retinomorphic application of a bilayer vertical OECT. Reproduced with permission [158]. Copyright 2024, Spring Nature.

OECTs have also been engineered to emulate the complex sensing of the human eye. Hu et al. developed a system for multicolor perception by integrating a stimuli‐responsive colored hydrogel with a photoactive Bi_2_S_3_ layer and a PEDOT:PSS channel [157], as depicted in Figure 11a. The hydrogel was synthesized via a horseradish peroxidase‐catalyzed colorimetric reaction and responds to different light by altering its color and transparency to modulate ion migration. The Bi_2_S_3_ layer provides broadband photoresponse, converting light into a driving photovoltage for ions, while the PEDOT:PSS channel supports mixed ion‐electron conduction. This design mimics the multicolor perception of human retinal cone cells. Additionally, fundamental synaptic plasticity functions, including PPF, spike number‐dependent plasticity (SNDP), and spike intensity‐dependent plasticity (SIDP), and the transition from STM to LTM can be achieved by adjusting the biomolecule concentration within the hydrogel. While a 4 × 4 array demonstrated successful color image memory, scaling this architecture remains challenging due to difficulties in maintaining uniform hydrogel thickness and consistent electrode contacts.

To increase integration density and functional complexity, researchers have explored new device geometries. Laswick et al. developed a vertical dual‐layer OECT with tunable antibipolar functionality [158], as shown in Figure 11b. By vertically stacking n‐type BBL and p‐type PEDOT:PSS, the researchers could tune the threshold voltage and current peaks via the thickness ratio of the layers. This architecture was used to construct circuits that mimic retinal signal pathways, encoding both wavelength and intensity. By connecting these devices in series or parallel, the system performed logic operations (AND, NOR, OR, NAND), effectively preprocessing signals in a manner analogous to retinal cones and rods. Furthermore, Xu et al. developed a novel optically readable OECT by integrating P3HT with an ionic gel electrolyte, which significantly enhances image features and image recognition accuracy [159].

Despite their promise, OECT‐based visual systems face inherent physical limitations. The reliance on ionic modulation introduces inherent temporal delays and nonlinearities, which can restrict the precision of synaptic weight updates. Additionally, the repeated trapping and detrapping of ions often induce mechanical swelling and material fatigue in the organic semiconductor, presenting a significant hurdle for the long‐term stability and durability of these devices.

#### 3.2.4 EGTs for Visual Perception Applications

EGTs are uniquely positioned for visual neuromorphic systems due to their superior ionic‐electronic coupling, which allows for the emulation of the complex biochemical signaling found in the biological retina and visual cortex. This section reviews recent progress in EGTs utilizing diverse material systems and electrolyte preparation methods to achieve advanced visual functionalities.

**FIGURE 12:**
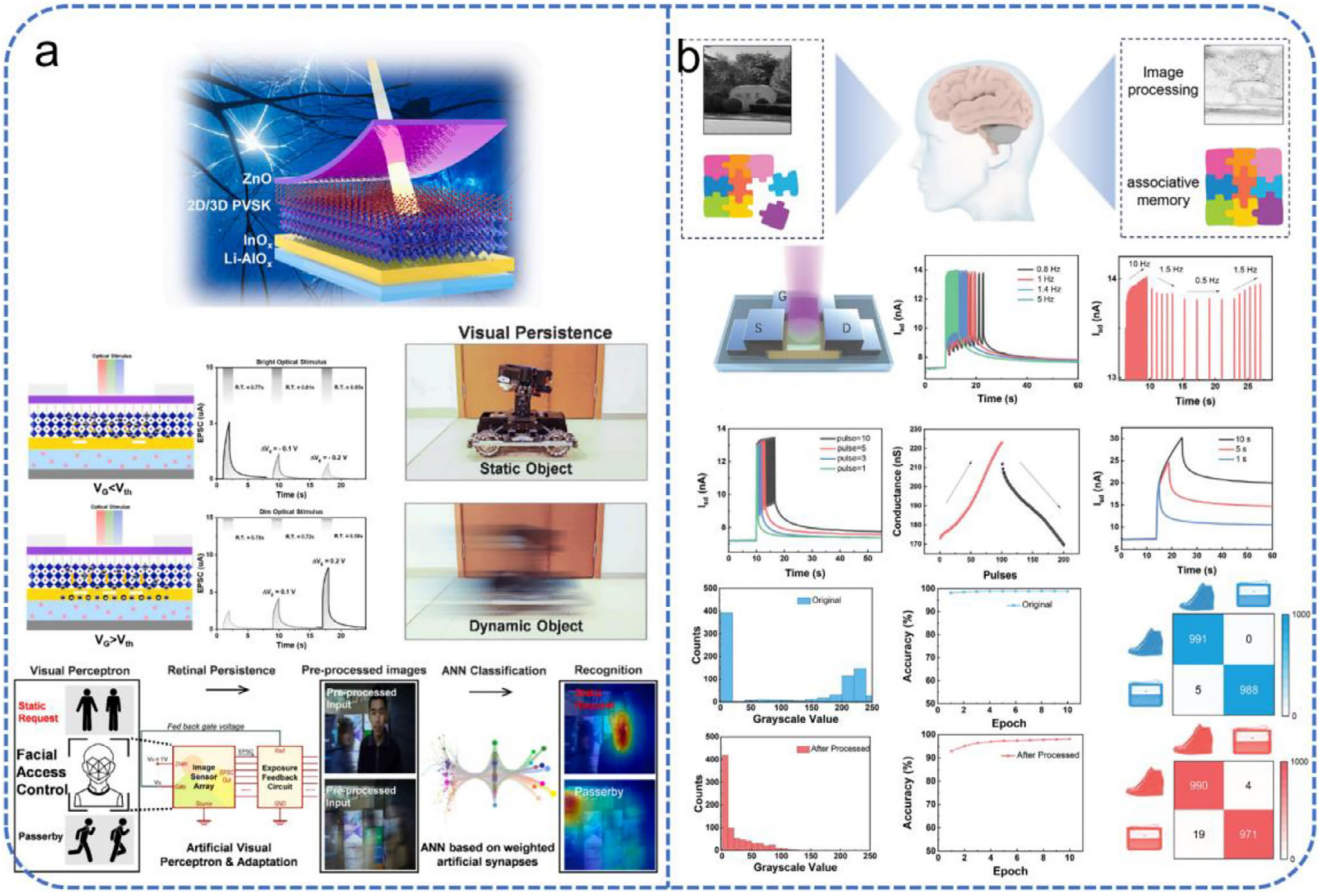
Schematic illustration of EGT‐based devices for neuromorphic visual perception. (a) Schematic of the solution‐processed heterojunction structure; array device demonstrating artificial visual adaptation and persistence; implementation in a cascaded facial access control system. Reproduced with permission [160]. Copyright 2022, Elsevier. (b) Guided by the BCM learning rule, the device implements pixel compression and associative memory learning. Reproduced with permission [162]. Copyright 2024, Wiley.

A primary focus in EGT research is the emulation of retinal adaptation and persistence. Liu et al. reported a hybrid‐dimensional heterojunction synaptic device combining a 2D/3D mixed perovskite (OAI‐FaPbI_3_) with an InO_x_ semiconductor channel, gated by a LiAlO_x_ electrolyte (Figure 12a) [160]. The LiAlO_x_ electrolyte facilitates lithium‐ion migration to modulate channel conductivity under electrical stimulation. Benefiting from the highly efficient ion regulation capability of the electrolyte layer, the device can adjust its photoresponse to varying light intensities, emulating both pupil adjustment and retinal neuron adaptation. Notably, the device demonstrates significant hysteresis during the processing of dynamic optical inputs, effectively emulating the biological phenomenon of visual persistence. In addition, A distinct negative photoresponse phenomenon was explored by Jin et al., using a side‐gated In_2_O_3_ transistor with an screen‐ printing Al_2_O_3_/ion‐gel gate stack [161]. By utilizing electrical pulses for charge trapping and light signals to reset conductance via the negative photoconductance (NPC) effect, this system adaptively tunes its perception threshold in response to ambient brightness, mimicking the light‐dark adaptation of the human eye.

Beyond front‐end adaptation, EGTs are highly effective at emulating experience‐dependent plasticity and high‐level cortical functions. Wang et al. demonstrated an IGZO‐based EGT capable of mimicking the Bienenstock–Cooper–Munro (BCM) learning rule (Figure 12b) [162]. In this device, optical illumination ionizes oxygen vacancies in the IGZO channel, altering carrier concentration in a history‐dependent manner. This results in a “sliding threshold” effect, where the synaptic weight update depends on the previous activity of the neuron. This mechanism enables sophisticated visual tasks such as edge detection and data compression, achieving a 98 % classification accuracy in pattern recognition. In a more bio‐sustainable approach, Serghiou et al. developed an EGT using an organic active layer (BuPTCD) and honey as a natural gate electrolyte [163]. The high viscosity and complex composition of honey result in slow ion migration, which enhances interaction efficiency with low energy. The device can distinguish and memorize different colors and light intensities, showing a response analogous to that of human retinal photoreceptors, while its biodegradable components offer a promising avenue for environmentally benign neuromorphic hardware.

Despite their promise, EGTs for visual perception face several challenges. Devices employing organic or moisture‐sensitive electrolytes often suffer from environmental degradation, limiting long‐term stability. Future research could prioritize advanced encapsulation techniques, material optimization, and the development of robust hybrid electrolytes. Furthermore, ionic crosstalk in dense arrays can lead to signal interference, constraining scalability. Implementing effective device isolation or crosstalk suppression architectures will be critical for realizing large‐scale, high‐fidelity visual neuromorphic systems.

#### 3.2.5 Arrays and Crossbar Architecture for Visual Perception Applications

While individual neuromorphic devices can emulate basic synaptic plasticity, the transition to high‐level visual perception requires the integration of these devices into dense, scalable architectures. Array integration leverages process compatibility to achieve high‐resolution image capture and parallel signal processing, facilitating a retina‐like mapping of visual information [164, 165, 166, 167]. In particular, the crossbar architecture, through its row‐column interconnections and capacity for parallel computation, facilitates feature extraction, pattern recognition, and event‐driven processing at the circuit level. Consequently, these three levels, from single devices to integrated arrays and finally crossbar systems, collectively form visual neuromorphic systems capable of parallel perception and brain‐inspired information processing.

**FIGURE 13:**
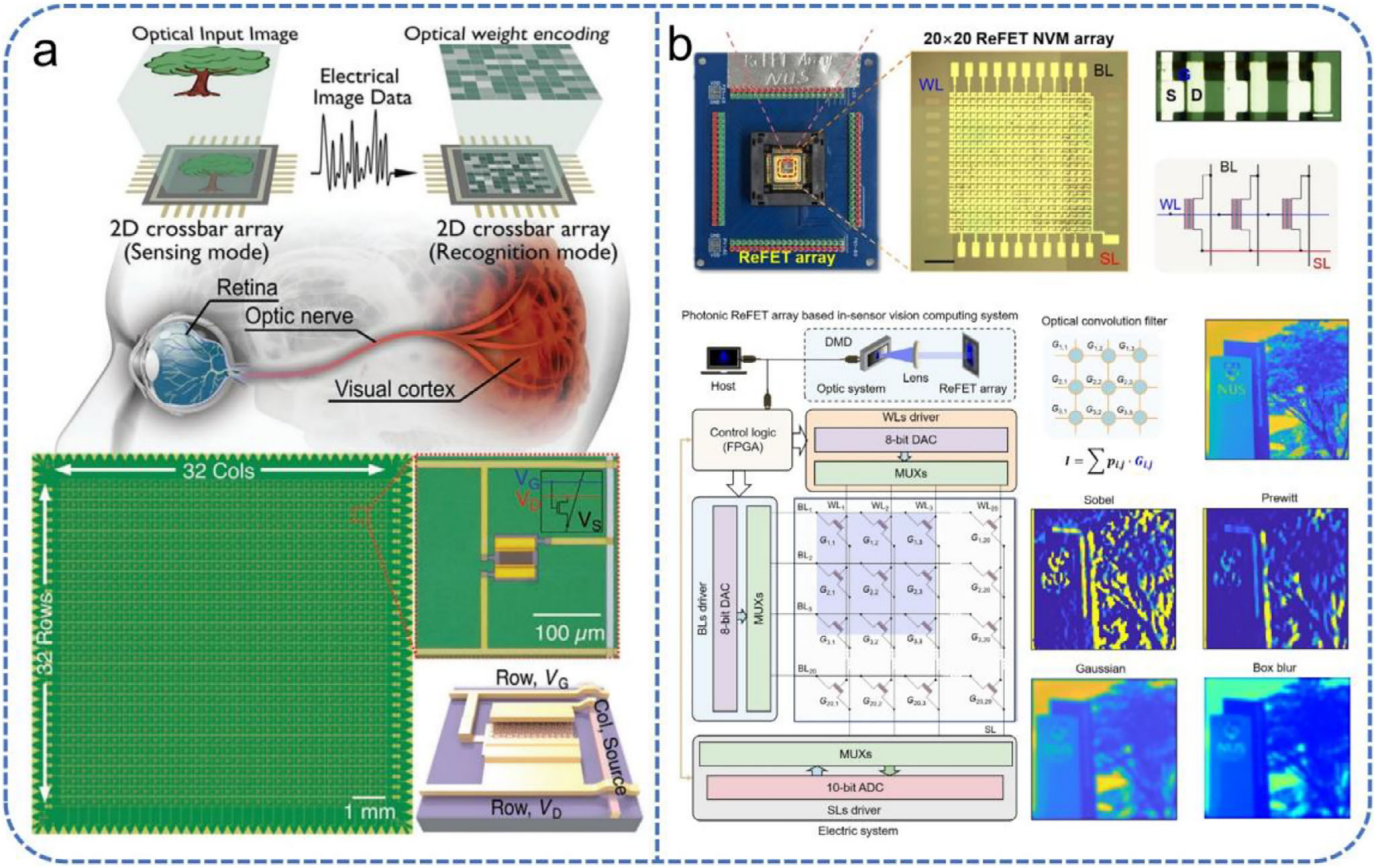
Schematic illustration of the array‐integrated crossbar architecture for neuromorphic visual perception. (a) Analog optoelectronic vision processor based on a MoS_2_ photo‐FET crossbar array. Reproduced with permission [166]. Copyright 2020, Wiley. (b) ReFET array based on an HZO/HSO superlattice and the in‐sensor vision computing system constructed from the ReFET array. Reproduced with permission [167]. Copyright 2025, Wiley.

A landmark implementation of this concept was reported by Jang et al., who proposed an atomically thin optoelectronic machine vision processor based on a crossbar array architecture (Figure 13a) [166]. This processor integrates visual perception and image recognition within a single platform using a 32 × 32 FET array. Functioning as an active pixel sensor array, it utilizes the photoconductivity effect of MoS_2_ to capture and store optical image data, achieving a spatial resolution far superior to conventional transition metal dichalcogenides‐based image sensors. In the front‐end sensing phase, the array performs full‐frame image acquisition by activating row lines and measuring column currents. For back‐end recognition, the same crossbar is reconfigured as an analog convolutional neural network, where the light‐programmed modulation of FET conductance simulates synaptic weights, enabling direct vector‐matrix multiplication for image classification. This architecture effectively integrates optoelectronic sensing and neural network computation within a platform, achieving 94 % accuracy in recognizing 1000 handwritten digits and significantly enhancing the functional complexity of integrated circuits.

Further advancing the paradigm of optical in‐memory computing, Figure 13b illustrates a 20 × 20 photonic neuromorphic platform based on IGZO‐FETs with superlattice gates [167]. This architecture achieves 272 stable conductance states (>8‐bit precision), enabling a high‐fidelity emulation of light‐dependent synaptic plasticity. For visual perception tasks, 3 × 3 sub‐arrays are configured as hardware convolutional kernels. By performing multiply‐accumulate operations directly between the incident light intensity and the pre‐programmed conductance states, the array extracts image features in the analog domain. This approach eliminates the need for the energy‐intensive analog‐to‐digital conversions and external multipliers required in conventional vision systems. When integrated into a visual transformer framework, this array achieved a 94.45 % classification accuracy on the Fashion‐MNIST dataset, demonstrating its potential for complex image recognition.

Despite these breakthroughs, several bottlenecks remain for large‐scale deployment. Fixed‐pattern noise, resulting from device‐to‐device variability and leakage currents within the crossbar, can severely limit the precision of parallel computations. Additionally, managing ionic or electrical crosstalk in high‐density multi‐layer arrays remains a significant engineering challenge. Future research must focus on enhancing fabrication uniformity and developing robust peripheral circuitry to suppress noise and leakage, thereby fully unlocking the potential of integrated visual neuromorphic systems.

### 3.3 FET‐Based Multisensory Integration for Neuromorphic Systems

Biological organisms rely on multisensory integration to navigate complex environments, synthesizing data from disparate modalities such as vision, audition, and somatosensation to form a robust, coherent perception [168, 169]. This integration enhances processing accuracy and sensitivity while reducing environmental uncertainty. For instance, while visual perception may be compromised in low‐light or occluded environments, the integration of complementary tactile or auditory cues ensures reliable environmental awareness. In neuromorphic hardware, FETs are uniquely suited for this task as their multi‐terminal architecture allows for the simultaneous modulation of channel conductance by diverse physical stimuli.

The fusion of visual and tactile signals typically requires the integration of flexible mechanoreceptors with photosensitive FETs. Tactile sensors such as triboelectric nanogenerators (TENGs), flexible ferroelectric electret nanogenerators (FENGs) [170], and piezoelectric nanogenerators (PENGs) [171] serve as artificial mechanoreceptors, converting pressure or touch into electrical signals. These signals can directly gate FETs to modulate their channel conductance. Concurrently, visual input is transduced by the photosensitive layer (e.g., quantum dots or photoconductive materials) of FETs, where absorbed photons generate electron–hole pairs that alter channel conductivity. The co‐processing of these signals enables the emulation of key integrative biological phenomena like reciprocal effectiveness and temporal congruency.

**FIGURE 14:**
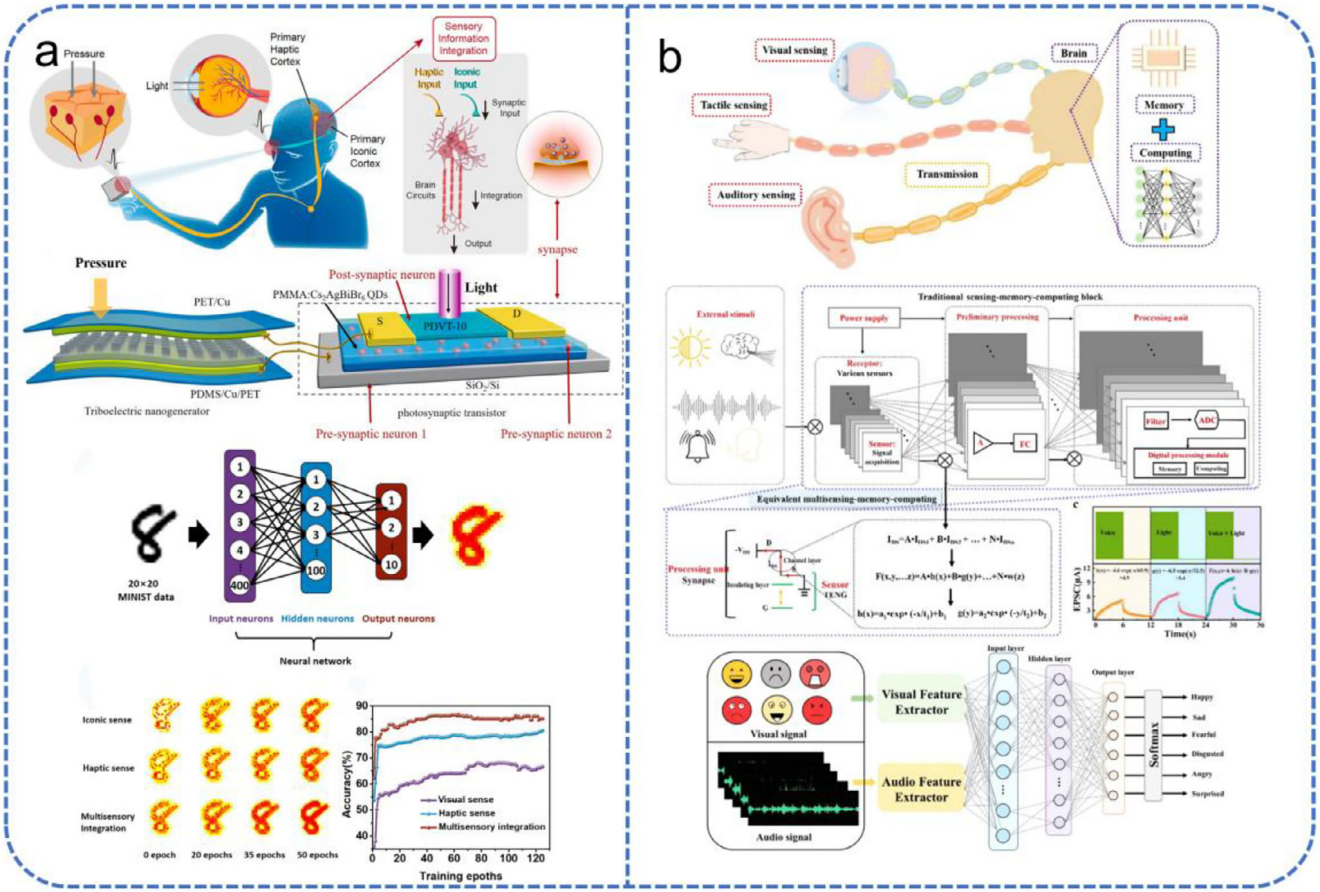
Multisensory perception system based on neuromorphic FETs. (a) Schematic of the visuotactile perception system, device response to light/force stimuli, and the corresponding ANN recognition rate vs. training epochs under unimodal and multimodal conditions. Reproduced with permission [172]. Copyright 2021, Elsevier. (b) An integrated framework for bio‐inspired multisensory processing. Reproduced with permission [175]. Copyright 2022, Springer Nature.

A representative implementation by Wu et al. utilized a micropatterned PDMS‐based TENG to gate a perovskite quantum dot‐modified FET (Figure 14a) [172]. The system employs a TENG as the tactile sensing unit, and its output voltage directly gates the transistor. The perovskite quantum dots function simultaneously as the charge‐trapping layer and photoresponsive materials. The electrical coupling of tactile and visual signals allows joint modulation of the channel current, successfully emulating synaptic plasticity and the principles of multisensory integration. The system achieved environment‐adaptive image recognition under extreme illumination, significantly enhancing recognition accuracy and image contrast. Inspired by the human auditory system, converting acoustic signals into electrical signals by neuromorphic hardware meets distinct challenges. Recently, several reports have addressed this issue through integrate auditor sensor with a synaptic device [173, 174, 175]. Liu et al. demonstrated that acoustic vibrations could induce periodic contact‐separation in a TENG, effectively transducing sound into electrical signals [174]. Although this early work did not integrate audition with vision, it laid the groundwork for subsequent multimodal systems. In a follow‐up study, the same group developed a self‐powered vertical tribo‐transistor by integrating a vertical FET with a TENG [175], as illustrated in Figure 14b. MXene nanosheets served a triple function as TENG top electrode, vertical FET source electrode, and light‐harvesting layer. This design enabled integrated tactile, auditory, and visual perception within a single device, and the visual + auditory model achieved a higher emotion recognition accuracy (94.05 %) than an individual model. Alternative encoding strategies have also been explored. Li et al. employed a Fourier transform‐based approach to convert acoustic signals into the frequency domain, encoding the spectral sequence into voltage pulse trains to modulate an EGT [119]. This device was also capable of optical sensing, thereby achieving reconfigurable audio‐visual perception within a single device.

Furthermore, the integration of vision with other senses, such as the vestibular system, is also important for advanced robotics and AI. An artificial motion‐sensory system combining an artificial vestibular unit with an artificial retina was proposed by Chen et al. [176]. This system replicated the biological principle of spatiotemporal congruency, improved motion recognition accuracy, and enabled rapid self‐protection. This study opened new possibilities for the integration of vision with other sensory systems.

Finally, a summary of recent advances in FET‐based visual perception systems, including materials, optical bandwidths, and synaptic plasticity ranges, is provided in Table 2. While most current studies focus on UV–vis spectral responses, future research should target infrared‐sensitive or full‐spectrum visual systems to broaden applicability and enable richer AI‐environment interaction. Meanwhile, developing low‐power FET‐based visual neuromorphic systems is essential for scalable, high‐throughput manufacturing techniques.

**TABLE 2:** A summary of structures, materials, optical response bandwidths, synaptic plasticity ranges, switching energies, and recognition accuracies of different FET‐based visual neuromorphic systems.

Category	Semiconductor layer materials	Optical response bandwidth (nm)	Synaptic plasticity range	Switching energy	Accuracy (MNIST)	Ref
FGFET	ReS_2_	532 nm	PPF/LTP/LTD/EPSC/PSC	28 pJ	98.15 %	[177]
	DPPDTT	405/532/635 nm	EPSC/ PPF/LTP/LTD	0.034 pJ	92.1 % (flat)/ 88.8 % (bending)/ 85.9 % (folding)	[91]
	IGZO	532 nm	PPF/STM/LTM/LTP/LTD	≈0.79 pJ	95.3 %	[178]
FeFET	a‐In_2_Se_3_	340/520/785830/940 nm	PPF/LTP/LTD	≈ 3.96 pJ	Fashion: 80 %/ Handwritten digit: 95 %/ Iris recognition: 97 %	[179]
	MoS_2_	450/532/650 nm	LTP/LTD/PPC	1.8 pJ	91 %	[180]
	a‐In_2_Se_3_	405/530/660 nm	PSC/PPF/STM/LTM	≈25 pJ	≈89 %	[181]
OECT	PEDOT:PSS	365 nm	EPSC/PPF/SRDP/SNDP/STM/LTM	≈90 µJ	/	[182]
	P3HT	450/550/670 nm	EPSC/IPSC/STM/LTM/PPF/PPD	/	88.86 %	[159]
	P3HT/Y6	365–850 nm	PPF/SIDP/SDDP/SNDP/STP/LTP	≈18 nJ	/	[183]
EGT	IGZO	450 nm	EPSC/STP/LTP/LTD	2.37 nJ	91.02 %	[184]
	ITO	365 nm	EPSC/STP/LTP/PPF	≈87.7 fJ	93.42 %	[185]
	BuPTCD	482/529/657 nm	STDP/PPF/PTP/ PPD/STP/LTP	2.4 pJ	/	[163]

## 4 Summary and Outlook

This review has systematically summarized the recent advances in FET‐based artificial visual neuromorphic systems. By leveraging diverse architectures—including FGFETs, FeFETs, OECTs, and EGTs—these systems have demonstrated remarkable capabilities in emulating the human visual pathway. Their low power consumption, high‐speed switching, and precise modulation of synaptic plasticity make them superior candidates for breaking the von Neumann bottleneck. While laboratory‐scale demonstrations have successfully realized functions such as edge detection, pattern recognition, and multisensory emotion analysis, transitioning these technologies into robust, real‐world applications in robotics, augmented reality, and bionic vision requires addressing several fundamental challenges.

### 4.1 Sensitivity and Response Speed

As core performance metrics, sensitivity and response speed determine how effectively neuromorphic devices process external stimuli. High sensitivity facilitates the detection of minute input fluctuations, enabling robust performance in weak‐light or high‐noise environments. Complementing this, high response speeds allow for the rapid perception of dynamic scenes, which is critical for the overall efficiency and precision of information processing. The sensitivity of devices to weak optical signals can be significantly enhanced through several strategies. These include utilizing materials with high absorption and conversion efficiency, such as MoS_2_ [186], graphene, and QDs. Additionally, utilizing semiconductors with high intrinsic mobility or optimizing the thickness of individual device layers can further improve charge carrier transport and the overall photoelectric response [187].

### 4.2 Long‐Term Stability and Robustness

These attributes are essential for the deployment of neuromorphic devices in real‐world applications. Stability ensures that the photoelectric response remains consistent during prolonged operation, whereas robustness refers to the device's ability to maintain functional integrity—such as synaptic weight modulation, signal transduction, and spike encoding—under varying environmental conditions. To this end, high‐performance materials like perovskites have been utilized to improve operational stability [78]. Furthermore, the integration of organic–inorganic composites [188] and self‐healing materials [189] provides a pathway to extend operational longevity and structural resilience. Such advancements enable visual perception systems to retain high‐precision sensing capabilities over extended periods.

### 4.3 High Integration Density and Scalability

Achieving high integration density and scalability is essential for overcoming von Neumann limitations. By embedding visual neuromorphic systems within a compact footprint, these systems facilitate parallel and localized data processing for large‐area, low‐cost, and bio‐integrated applications. This is typically pursued across three hierarchical levels: device, circuit, and system. At the device level, scaling down channel lengths and reducing layer thicknesses directly enhances integration density and operating speeds while minimizing power consumption. At the circuit level, high‐density arrays and optimized architectures are critical for accelerating signal transmission and mitigating crosstalk in practical applications. At the system level, the development of in‐sensor computing and multi‐modal integration—utilizing array‐based organic semiconductor architectures or in‐memory computing designs—allows for the emulation of complex brain‐like perception and high‐efficiency decision‐making. Furthermore, maintaining uniform performance at this scale requires material systems compatible with wafer‐scale fabrication and device architectures tolerant to process variations [190]. Therefore, the development of advanced techniques for precise photolithographic patterning or uniform solution‐based processing is crucial for achieving high‐density integration with excellent uniformity and scalability [191].

In summary, the synergy of material innovation, device‐level optimization, and hierarchical system design will pave the way for a new generation of energy‐efficient, bio‐inspired visual neuromorphic systems. These advancements will redefine the capabilities of flexible and wearable electronics, also providing the essential hardware foundation for the next era of autonomous robotics and intelligent sensing [192].

## Conflicts of Interest

The authors declare no conflicts of interest.

## Data Availability

The authors have nothing to report.
